# Summary of the National Advisory Committee on Immunization (NACI) statement update on the recommended use of palivizumab to reduce complications of respiratory syncytial virus infection in infants

**DOI:** 10.14745/ccdr.v48i78a08

**Published:** 2022-07-07

**Authors:** Dorothy Moore, Angela Sinilaite, April Killikelly

**Affiliations:** 1 NACI RSV Working Group Chair; 2 Centre for Immunization Readiness, Public Health Agency of Canada, Ottawa, ON

**Keywords:** NACI, National Advisory Committee on Immunization, guidance palivizumab, PVZ, respiratory syncytial virus, RSV

## Abstract

**Background:**

Respiratory syncytial virus (RSV) is the most common cause of lower respiratory tract infection in young children worldwide. Underlying health conditions, especially premature birth, chronic lung disease and congenital heart disease, predispose to severe RSV illness. The only means of prophylaxis against RSV disease is passive prophylaxis with the monoclonal antibody, palivizumab (PVZ) (Synagis^TM^). The National Advisory Committee on Immunization (NACI) published a statement for PVZ use in 2003. The purpose of this article is to update previous NACI recommendations for the use of PVZ, taking into consideration recent data on RSV burden of illness, effectiveness of PVZ in infants at risk of more severe RSV disease and economic implications of PVZ use.

**Methods:**

The NACI Working Group and external experts performed systematic literature reviews on three topics to support updated NACI guidance: 1) RSV burden of disease; 2) PVZ effectiveness; and 3) cost effectiveness of PVZ prophylaxis. Full details and results are presented in the statement and supporting documents.

**Results:**

Respiratory syncytial virus hospitalization (RSVH) rates are highest in children younger than one year of age and especially in the first two months of life. In various populations of infants at risk of severe RSV infection, PVZ prophylaxis is associated with reductions of 38%–86% in the risk of RSVH. Only rare cases of anaphylaxis have been reported after decades of use. Palivizumab is expensive and only cost-saving in rare scenarios.

**Conclusion:**

Updated NACI recommendations on use of PVZ for the prevention of complications of RSV in infants are now available.

## Introduction

Respiratory syncytial virus (RSV) is the most common cause of lower respiratory tract infection in young children worldwide. It causes yearly outbreaks of respiratory tract disease; in Canada from late fall to early spring. While many infections are simple colds, children younger than two years of age are at risk of severe disease such as bronchiolitis or pneumonia and may be hospitalized. Underlying health conditions, especially premature birth, chronic lung disease and congenital heart disease predispose to more severe RSV illness. Reinfections occur throughout life as infection produces only partial and temporary immunity, although reinfections are usually milder than the initial one. At present, there is no vaccine available to prevent RSV; the only means of prophylaxis against RSV disease is temporary passive protection with the monoclonal antibody preparation, palivizumab (PVZ) (Synagis^TM^).

In 2003, the National Advisory Committee on Immunization (NACI) published recommendations on the use of PVZ for the prevention of RSV disease ((1)). At that time, NACI recommended PVZ be used during the RSV season for premature infants (younger than or equal to 32 weeks gestational age [wGA] who would be younger than six months of chronological age at the start of RSV season), children younger than 24 months of age with chronic lung disease of prematurity requiring oxygen and/or medical therapy in the previous six months or other pulmonary disorders requiring oxygen therapy, and children younger than 24 months of age with hemodynamically significant congenital heart disease. Palivizumab prophylaxis could also be considered for children born at younger than 35 wGA who are younger than six months of age at the start of RSV season and who live in remote northern communities ((1)).

The purpose of this article is to update previous NACI recommendations for the use of PVZ, taking into consideration recent data on burden of illness due to RSV disease, the efficacy and effectiveness of PVZ in infants at risk of more severe RSV disease and the economic implications of PVZ use.

Details can be found in the updated NACI Advisory Committee statement: *“Recommended use of palivizumab to reduce complications of respiratory syncytial virus infection in infants”* ((2)).

To support this work, the Working Group and other expert bodies performed three systematic literature reviews using standard NACI methodology: the burden of RSV disease in young children in high-income countries comparable to Canada (published in September 2021) ((3)); the effectiveness of PVZ prophylaxis on reducing the complications associated with RSV in infants (results summarized in the statement and full details to be published as a separate document) ((4)); and the cost-effectiveness of PVZ prophylaxis for RSV (results summarized in the statement and full details to be published as a separate document) ((5)).

The respiratory syncytial virus hospitalization (RSVH) rates are highest in children younger than one year of age and especially in the first two months of life. Prematurity is associated with greater risk for RSVH, longer hospital length of stay and a higher rate of admission to an intensive care unit. Children younger than two years of age with chronic lung disease of prematurity or younger than one year of age with hemodynamically significant congenital heart disease are also at greater risk for RSVH. Those with cystic fibrosis, Down syndrome and immunodeficiency may also be at increased risk. High rates of hospitalization for RSV have been reported in term infants living in some remote indigenous communities. Respiratory syncytial virus hospitalization in infancy may be associated with greater wheeze and asthma medication use in early childhood, but RSV causation has not been established.

Palivizumab has only been studied in children younger than two years of age with underlying health conditions, with the exception of one recent study of healthy term Inuit infants residing in remote northern communities. In various populations of infants at risk of severe RSV infection, PVZ prophylaxis is associated with reductions of 38%–86% in the risk of RSVH, with number of treatments needed to treat to prevent one hospitalization of 2 to 54. Reductions in RSVH of 38%–80% for premature infants, 39%–86% for those with chronic lung disease of prematurity, and 45%–51% for those with hemodynamically significant congenital heart disease have been reported. Recommendations for other groups considered to be at equivalent risk of severe RSV disease are based on extrapolations from these data.

A previous Canadian Immunization Guide recommendation that PVZ prophylaxis should be considered for all Inuit children in northern remote communities who are younger than six months of age at the start of RSV season, regardless of gestational age, was reassessed. NACI now recommends that PVZ should not be offered routinely to healthy term infants living in remote northern Inuit communities but may be considered for such communities if the documented RSVH rate for term infants is very high. This change was based on the limited evidence available, including one study showing no effect of PVZ prophylaxis on RSVH in healthy full-term infants living in a northern Inuit population in one region of Canada with a RSVH rate for all infants younger than one year of age of 5%, and a qualitative study in that same population that identified significant acceptability and feasibility issues with PVZ prophylaxis.

Palivizumab has been used for over two decades in many countries and has a good safety record, with very rare cases of anaphylaxis being the major serious adverse event. Palivizumab is expensive, with incremental effectiveness ratios per quality-adjusted life year estimated from less than $1,000 to over $2M in various scenarios. In various high-risk groups, 64%–100% of estimates were less than $50,000 per quality-adjusted life year. In rare scenarios it may be cost saving.

The key recommendations are summarized below.

## 2022 NACI recommendations for use of palivizumab to reduce complications of respiratory syncytial virus infection in infants

- Palivizumab **should be offered** to premature infants of younger than 30 wGA and younger than 6 months of age at onset of or during the RSV season; children aged younger than 24 months with chronic lung disease of prematurity who require ongoing oxygen therapy within the six months preceding or during the RSV season; infants aged younger than 12 months with hemodynamically significant congenital heart disease; and infants born at younger than 36 wGA and age younger than six months old living in remote northern Inuit communities who would require air transport for hospitalization. For children with both hemodynamically significant congenital heart disease and chronic lung disease, recommendations for chronic lung disease should be followed.
- Palivizumab **may be considered** for premature infants of 30–32 wGA and age younger than three months who are at high risk for exposure to RSV; selected children younger than 24 months of age with severe chronic lung disease due to cystic fibrosis or other etiology who require ongoing oxygen therapy or assisted ventilation in the six months preceding or during the RSV season; infants younger than 12 months of age with hemodynamically significant chronic cardiopathy other than congenital; children aged 12–24 months awaiting heart transplant or having received a heart transplant within six months of onset of the RSV season; and children aged younger than 24 months with severe immunodeficiency. It may also be considered for term infants aged younger than six months living in remote Inuit communities with very high rates of hospitalization for RSV among term infants and for infants of younger than 36 wGA and age younger than six months living in other remote communities with high rates of hospitalization for RSV and where air transport would be required for hospitalization.
- Palivizumab **should not be offered** to otherwise healthy infants born at or after 33 wGA; or to siblings in multiple births who do not otherwise qualify for prophylaxis. It should not be offered **routinely** for children younger than 24 months of age with cystic fibrosis; for children younger than 24 months of age with Down syndrome without other criteria for PVZ; or for healthy term infants living in remote northern Inuit communities unless hospitalization rates for RSV are very high. It should not be used for the prevention of recurrent wheezing or asthma in the absence of other indications.
- Palivizumab **should not be given** to prevent hospital-associated RSV infection in eligible children who remain in hospital. It **may be considered** when all other measures have failed to control an RSV outbreak in a neonatal intensive care unit.
